# Schistocyte quantitation, thrombotic microangiopathy and acute kidney injury in Australian snakebite coagulopathy [ASP28]

**DOI:** 10.1111/ijlh.13497

**Published:** 2021-02-22

**Authors:** Tina Noutsos, Bart J. Currie, Simon G. Brown, Geoffrey K. Isbister

**Affiliations:** ^1^ Menzies School of Health Research Charles Darwin University Darwin NT Australia; ^2^ College of Medicine and Public Health Flinders University Adelaide SA Australia; ^3^ Division of Medicine Royal Darwin Hospital Darwin NT Australia; ^4^ Centre for Clinical Research in Emergency Medicine University of Western Australia Perth WA Australia; ^5^ Aeromedical and Medical Retrieval Division Ambulance Tasmania Hobart TAS Australia; ^6^ Clinical Toxicology Research Group University of Newcastle Newcastle NSW Australia

**Keywords:** acute kidney injury, hemolysis, schistocytes, snakes, thrombotic microangiopathies

## Abstract

**Introduction:**

The major systemic manifestation of hemotoxicity in human snakebite envenoming is venom‐induced consumption coagulopathy (VICC). A subset of patients with VICC develop thrombotic microangiopathy (TMA), in which acute kidney injury (AKI) occurs. We aimed to investigate the association between schistocytosis in snakebite patients with VICC and AKI, compared to non‐envenomed patients.

**Methods:**

Serial blood films collected from a prospective cohort of snakebite patients (Australian Snakebite Project) were examined. Cases were classified a priori as non‐envenomed snakebites (normal controls), envenomed without VICC, partial VICC without AKI, complete VICC without AKI, and VICC with AKI based on defined clinical and laboratory criteria. The percentage of schistocytes between groups was compared and correlated by Kendall's tau *b* test.

**Results:**

Seven hundred and eighty blood films from 234 snakebite cases were analyzed. There was a statistically significant correlation (*τ* = .69, SE .03, *P* < .001) for schistocytosis between the ordered groups of non‐envenomed snakebites, envenomed without VICC, partial VICC without AKI, complete VICC without AKI, and VICC with AKI groups. Patients with VICC and AKI had a platelet nadir median of 42 × 10^9^/L (interquartile range [IQR] :25‐130 × 10^9^/L), hemoglobin nadir of median 107 g/L (IQR 66‐122 g/L), and maximum LDH median of 1128 U/L (IQR 474‐3255 U/L). A 1.0% threshold for schistocytosis yielded 90% sensitivity (95% CI: 67%‐98%) and 71% specificity (95% CI: 62%‐79%) for predicting AKI in patients with VICC.

**Conclusion:**

Schistocyte quantitation has good diagnostic utility in snakebite patients with VICC. A definition of snakebite TMA as MAHA with ≥1.0% schistocytes and thrombocytopenia, would appear to be appropriate.

## 1 INTRODUCTION

Venomous snakebite is a significant global health problem, causing a range of different clinical toxin syndromes. Snakebite is mainly an issue in rural and remote impoverished tropical and sub‐tropical regions of the world.^1^ Whilst bites from venomous snakes may not result in systemic poisoning (envenoming), when envenoming does occur it is associated with significant morbidity and mortality.^2^ In addition to neurotoxicity, myotoxicity, and cytotoxicity, the heart and blood are commonly affected in snake envenoming, with resultant clinical scenarios including hypotension, cardiovascular collapse, and coagulopathy.

The commonest form of coagulopathy is venom‐induced consumption coagulopathy (VICC). VICC is associated with a variety of hemotoxins in corresponding snake species' venom.^3^ Hemotoxic venoms may act as thrombin‐like enzymes, prothrombin activators, or other clotting factor activators including factor V, X and plasminogen.^3^, ^4^ VICC presents with prolonged clotting times and hypofibrinogenemia and recovers at a rate consistent with neutralization of the venom and synthesis of new clotting factors.^4^ The main complication of VICC is bleeding, which can be life‐threatening.^3^

A subset of cases of snakebite with VICC develop an additional different clinicopathological syndrome: thrombotic microangiopathy (TMA), which is predominantly associated with acute kidney injury (AKI).^5^, ^6^, ^7^, ^8^, ^9^, ^10^ In contrast to VICC, snakebite TMA is marked by a delayed thrombocytopenia and microangiopathic hemolytic anemia (MAHA), presenting with schistocytes on the peripheral blood film.^7^ Multiple small observational pathological studies of snakebite and AKI have reported snakebite cases with schistocytes on the peripheral blood film, together with histological findings of TMA in the kidney.^6^, ^7^, ^11^, ^12^

TMAs more broadly are a diverse range of disorders defined by clinicopathological criteria, including blood film examination for red cell schistocytes, which are the red cell morphological hallmark of TMA. Schistocytes form due to mechanical damage to red blood cells, and they are defined morphologically by sharp rectilinear fracture or tear lines and sharp angles. The occurrence of schistocytosis has been best studied in well elucidated TMA disorders including thrombotic thrombocytopenic purpura (TTP) and hemolytic uremic syndrome (HUS). A schistocytosis threshold of 1% has been cited as a robust cut‐off for the presence of TMA on a blood film, and in TTP and HUS it is often much higher than this.^13^, ^14^ Normal blood films show only very rare schistocytes, typically less than 0.2%‐0.5%.^13^, ^15^, ^16^

There are no comprehensive studies examining blood film findings in snakebite VICC or TMA, nor a standardized or consistent definition of snakebite‐associated TMA. Laboratory features of snakebite‐associated TMA including blood film schistocytes have to date been reported in mostly case reports, small case series and occasional larger observational studies.^5^, ^6^, ^17^, ^18^, ^19^, ^20^ None have systematically examined blood film findings in a quantitative or comparative way between clinical toxin syndromes of snakebite. Confounding this, the scientific literature both VICC and TMA in snakebite have historically been described by different and sometimes unclear or erroneous nomenclature, including defibrination syndrome, intravascular hemolysis, or disseminated intravascular coagulation (DIC); variably in association with AKI.^17^, ^21^, ^22^ VICC and snakebite associated TMA are distinctly different from DIC with respect to etiology, presenting features, and outcomes. DIC is an acquired thrombo‐hemorrhagic syndrome associated with widespread activation of the tissue factor/factor VIIa pathway.^23^, ^24^ It is strongly associated with the systemic inflammatory response syndrome in sepsis, severe trauma, burns, and obstetric calamities. Compared to the hemorrhagic risk in VICC, and the renal end‐organ injury of snakebite‐associated TMA, DIC is marked by multiorgan dysfunction syndrome, which confers a high mortality rate.^17^ Much of the existing literature referencing snakebite and “defibrination syndrome,” “intravascular hemolysis,” or DIC is difficult to interpret given the ill‐defined nomenclature typically used.^17^

We report a prospective cohort of snakebites from the Australian Snakebite Project (ASP), with quantitation of schistocytes on serial blood films using the International Council for Standardization in Haematology (ICSH) method. Our primary aim was to investigate the association between blood film schistocyte counts in snakebite patients, and VICC with and without AKI, compared to non‐envenomed patients. In addition, we aimed to determine a schistocyte cut‐off suggestive of TMA in snakebite patients.

## 2 MATERIALS AND METHODS

The ASP is a multicenter prospective cohort study, enrolling patients with suspected or definite snakebites at time of presentation to a participating hospital. Human research ethics committee (HREC) approval has been obtained from major State and Territory HRECs, including the Hunter New England HREC and the University of Newcastle (07/11/21/3.06), the HREC of the Northern Territory Department of Health and Menzies School of Health Research (04/08), the Royal Perth Hospital Ethics Committee and South Metro Area Health Service (RA‐08/003), Western Australian Country Health Service Ethics Committee (2008:03, REC200835), Tasmania Network (H00109965), Gold Coast Health Service District HREC as well as an additional ten HRECs responsible for all facilities involved. Informed consent is obtained from all patients or from a parent/guardian for children (<18 years). Children aged under two years of age are not included.

Patients are recruited to ASP from referrals by emergency departments, clinical toxicologists and staff from the Australian Poisons Information Centre Network. Patient information sheets and consent forms are sent to the referring clinicians. Data is collected on purpose designed datasheets, including demographics, circumstances of bite, clinical effects, pathology results, length of stay, treatment (antivenom), and complications (acute kidney injury, death). At enrollment, serial blood samples are taken including a full blood count, peripheral blood film, coagulation studies and biochemistry at initial presentation, 2 hours, 6 hours and then 24‐hourly until time of hospital discharge. All data is entered in a relational database and any missing data is extracted from patient medical records.

The database is reviewed by the chief investigator and ASP cases are defined and classified by snakebite clinical envenoming syndrome, prior to blood film examination by the investigators. Classifications are non‐envenomed suspected or confirmed snakebite (negative normal controls); envenomed without VICC; envenomed with partial VICC and no AKI; envenomed with complete VICC and no AKI; and envenomed with VICC and AKI. Complete and partial VICC were classified by INR, fibrinogen and D‐dimer testing of all snakebite cases, as previously defined.^4^, ^25^, ^26^ Complete VICC was defined as an undetectable fibrinogen by Clauss method; OR INR >3 and D‐dimer at least 10 times assay upper limit of normal or >2.5 mg/L. Partial VICC was defined as a prolonged INR <3 and raised D‐dimer, and low but recordable fibrinogen. AKI was defined and classified a priori by the 2012 Kidney Disease: Improving Global Outcomes (KDIGO) classification.^27^

Serial peripheral blood films collected from ASP cases from 2005 to 2014 at initial presentation, 2 hours, 6 hours, and then 24 hourly, were examined and a quantitation of red cell schistocytes was performed. Blood films were prepared from EDTA blood samples and spread by the attending hospital and then forwarded to the research center. Films were stained via an automated slide stainer using routine Romanowsky method. Blood films were examined in random order blinded to patient identity and diagnosis. For each blood film, 1000 erythrocytes were counted and examined at high power by oil immersion (100× objective, magnification ×1000). All blood films were examined by the first author (TN). A total schistocyte percentage was calculated as per the ICSH method.^28^

Quality control was ensured by exclusion criteria for rejection of a slide if poor film quality was present, defined by either insufficient working area due to an improperly or poorly spread film such that 1000 cells could not be counted in the working area of the slide; unacceptable morphology within working area (for example presence of artefact such as crenation of cells, staining or water artefact from poor fixation); or the presence of significant global red cell poikilocytosis (for example thalassemia major, other haemoglobinopathies, significant dysplasia) making counting of schistocytes difficult or invalid.

Schistocyte quantitation was presented for the different clinical toxin syndromes by Tukey box plots for median, interquartile range (IQR), 95% percentiles and outliers. Statistical analysis for correlation and comparison between ordered groups (non‐envenomed, envenomed with no VICC, partial VICC with no AKI, complete VICC with no AKI, and VICC with AKI, including AKI KDIGO 1, 2 and 3 ordinal groups) was performed using Kendall's tau *b* test for non‐parametric data. Sensitivity and specificity were calculated for VICC with AKI vs VICC with no AKI (negative controls) at the ICSH recommended cut‐off of 1.0% for significant schistocytosis. A *P* value of <.05 was set for significance. Receiver Operating Characteristic (ROC) curves were plotted as follows. Sensitivity and specificity of the results using quantitations for schistocytes using the ICSH method were performed for a range of different cut‐off thresholds including around the previously reported cut‐off of 1.0% for a positive schistocyte quantitation test. The Youden Index (*J*) was calculated to measure the clinical diagnostic ability of schistocyte quantitation, where *J* = max(*c*)[Sensitivity(*c*) + Specificity(*c*) − 1], with *c* the cut‐off. ROC curves were used to determine the utility of quantitation of schistocytosis as a diagnostic test for differentiating the clinical toxin syndromes of VICC with no AKI from VICC with AKI. The comparison of VICC with no AKI vs VICC with AKI was chosen a priori, given that TMA occurs only in the context of VICC, and can occur both in partial VICC and complete VICC. The diagnostic utility of schistocyte quantitation lies in the predictive validity on differentiating these two classifications.

Statistical analysis was conducted by Graphpad prism software version 8.4.2 for Windows, GraphPad Software, www.graphpad.com; with the exception of Kendall's tau *b* test which was performed in IBM SPSS Statistics version 25.0 for Windows.

## 3 RESULTS

**FIGURE 1:**
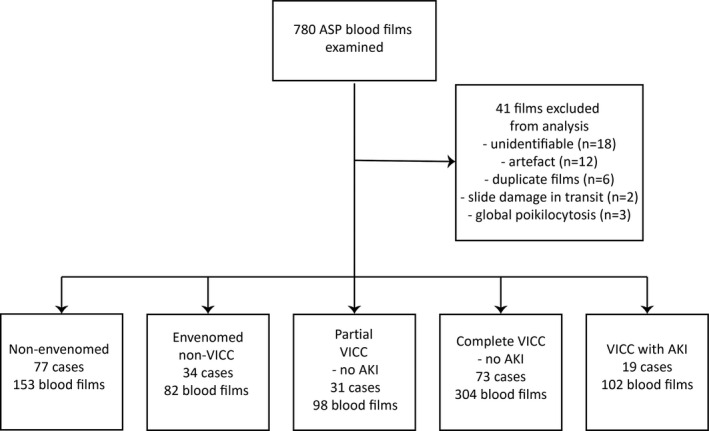
Classification of blood films from snakebite cases from the Australian Snakebite Project. AKI, acute kidney injury; ASP, Australian Snakebite Project; TMA, thrombotic microangiopathy; VICC, venom‐induced consumption coagulopathy

Seven‐hundred‐and‐eighty peripheral blood films were examined for 234 snakebite patients in total. Forty‐one blood films were excluded from the analysis due to poor quality or inadequate patient identifiers. This left 739 blood films for analysis, which included 102 blood films from 19 cases with VICC (partial or complete) and AKI; 304 blood films from 73 cases with complete VICC but no AKI; 98 from 31 cases with partial VICC; 82 from 34 cases with envenoming but no VICC or TMA and 153 films from 77 non‐envenomed cases. (Figure 1).

Most cases were adults (n = 191, 82%), and predominantly male (n = 173, 74%). Envenomed non‐VICC cases were predominantly due to red‐bellied black snake envenoming (n = 21, 62%). The majority of cases with VICC and no AKI were from brown snake (n = 57, 55%), tiger or tiger group (n = 24, 23%), rough‐scaled snake (n = 13, 13%), and taipan (n = 5, 5%) envenoming. The majority of cases with VICC and AKI were brown snake envenoming (n = 11, 58%) (Table 1).

**TABLE 1:** Baseline characteristics of included patients

	Non‐envenomed (n = 77)	Envenomed non‐VICC (n = 34)	Partial VICC no AKI (n = 31)	Complete VICC no AKI (n = 73)	VICC with AKI (n = 19)
Sex, n (%)					
Male	53 (69)	24 (71)	27 (87)	54 (74)	15 (79)
Female	24 (31)	10 (29)	4 (13)	19 (26)	4 (21)
Age (y) median (IQR)	41 (24‐55)	33 (17‐52)	34 (21‐53)	38 (24‐52)	54 (46‐60)
Snake species, n (%)					
Brown (*Pseudonaja* spp.)	7 (9)	1 (3)	9 (29)	48 (66)	11 (58)
Tiger (*Notechis scutatus*)	3 (4)		9 (29)	12 (16)	2 (11)
Tiger group	—	1 (3)	1 (3)	2 (3)	—
Red bellied black snake (*Pseudechis porphyriacus*)	3 (4)	21 (62)	—	—	—
Black bellied swamp snake (*Hemiaspis signata*)	1 (1)	—	—	—	—
Taipan (*Oxyuranus* spp.)	1 (1)	—	4 (13)	1 (1)	5 (26)
Slaty gray snake (*Stegonotus cucullatus*)	1 (1)	—	—	—	—
Rough‐scaled snake (*Tropidechis carinatus*)	—	—	5 (16)	8 (11)	—
Stephens banded snake (*Hoplocephalus stephensii*)	1 (1)	—	—	—	—
Mulga snake (*Pseudechis australis*)	1 (1)	5 (15)	—	—	—
Death adder (*Acanthophis antarcticus*)	—	5 (15)	—	—	—
Pale headed snake (*Hoplocephalus bitorquatus)*	—	—	1 (3)	1 (1)	—
Sea snake	—	1 (3)	—	—	—
Whip snake (*Demansia* spp.)	2 (3)	—	—	—	—
Unknown/nil	57 (74)	—	2 (6)	1 (1)	1 (5)
Platelet nadir ×10^9^/L median (IQR)	218 (197‐275)	214 (191‐247)	206 (133‐235)	157 (115‐199)	42 (25‐130)
Hemoglobin nadir g/L median (IQR)	136 (125‐144)	134 (129‐144)	131 (125‐141)	123 (110‐132)	107 (66‐122)
LDH maximum U/L median (IQR)	206 (170‐230)	218 (175‐268)	247 (214‐359)	344 (279‐434)	1128 (474‐3255

Abbreviations: AKI, acute kidney injury; IQR, interquartile range; LDH, lactate dehydrogenase; VICC, venom induced consumption coagulopathy.

Patients with VICC and AKI typically developed thrombocytopenia and anemia, with a platelet nadir median of 42 × 10^9^/L (IQR 25‐130 × 10^9^/L), and hemoglobin nadir median of 107 g/L (IQR 66‐122 g/L). Maximum LDH for the VICC with AKI group was a median of 1128 U/L (IQR 474‐3255 U/L) (Table 1). Of the 19 cases with VICC and AKI, 13 (68%) had complete VICC and 6 (32%) had partial VICC; one (5%) had KDIGO stage 1 AKI, eight (4%) stage 2 AKI, and 10 (53%) stage 3 AKI. There were no cases with AKI in either the non‐envenomed or envenomed non‐VICC groups (Table 1).

**FIGURE 2:**
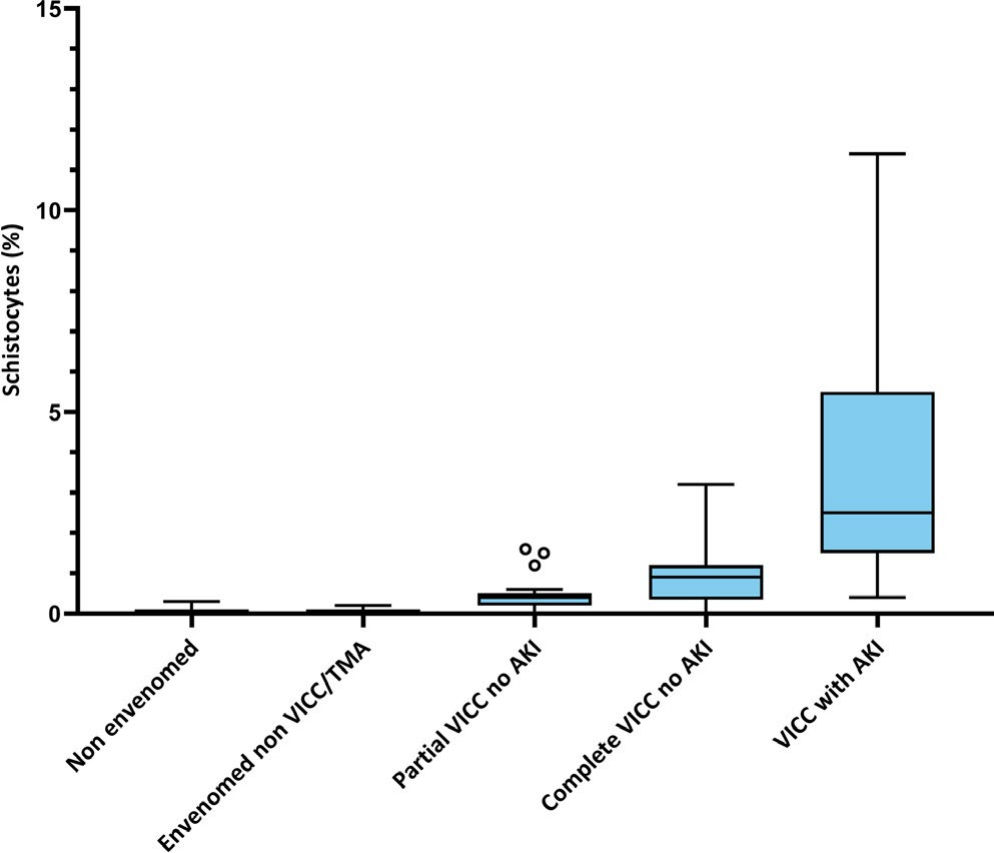
Box and whisker plot of peak schistocyte quantitation using ICSH method by clinical toxin syndrome (Tukey plot; boxes indicate medians with interquartile ranges, whiskers indicate 5 and 95% percentiles, and **ο** indicate outliers). AKI, acute kidney injury; TMA, thrombotic microangiopathy; VICC, venom‐induced consumption coagulopathy

There was an exponential increase in the peak schistocyte count for the five groups in order: non‐envenomed, envenomed without VICC, partial VICC without AKI, complete VICC without AKI, and VICC with AKI (Figure 2). Non‐envenomed and envenomed cases without VICC had minimal schistocytosis, with both groups median 0.0% (IQR 0.0%‐0.1%); and 95% CI of the median 0.0%‐0.0% and 0.0%‐0.1% respectively. The partial VICC without AKI group had a schistocyte peak of median 0.4% (IQR 0.2%‐0.5%). The complete VICC without AKI group had a partial overlap with the ICSH recommended 1.0% threshold for significance for TMA, with median peak 0.9% (IQR 0.4%‐1.2%). The VICC with AKI group had a peak schistocyte quantitation significantly above the 1.0% threshold, median 2.5% (IQR 1.5%‐5.5%). There was a significant correlation for the proportion of schistocytes by ICSH method for both the five ordered groups from non‐envenomed to VICC with AKI (Kendall's tau *b* correlation coefficient .69, Standard error [SE] .03, *P* < .001); and for the three ordered groups of partial VICC without AKI, complete VICC without AKI, and VICC with AKI (Kendall's tau *b* correlation coefficient .48, SE .06, *P* <.001).

Calculating the Youden index gave an optimum threshold of 1.15% schistocytes, with a *J* value of 0.68. The recommended ICSH schistocyte quantitation cut‐off of 1.0% for TMA gave a 90% sensitivity (95% CI: 67%‐98%) and 71% specificity (95% CI: 62%‐79%) for a diagnosis of VICC with AKI (n = 19) vs VICC without AKI (n = 104) (Figure S1, Table S1). A schistocyte cut‐off of 1.0% together with thrombocytopenia (platelets < 150 × 10^9^/L) gave a 79% sensitivity (95% CI: 56%‐92%) and 86% specificity (95% CI: 77%‐91%) for the diagnosis of VICC with AKI vs VICC without AKI. Of the 19 cases with VICC and AKI, 15 (79%) presented with thrombocytopenia and MAHA (defined by anemia with schistocytes ≥1.0%); two (11%) with MAHA without thrombocytopenia, and two (11%) with thrombocytopenia and mild anemia with <1.0% schistocytes. Of these two outliers, both were taipan envenomings with complete VICC, and AKI KDIGO 2 (n = 1) and 3 (n = 1). Both cases only had blood films available for examination from the same day of snakebite and were un‐labeled with respect to time of collection with no blood films available at later timepoints. For the 19 cases with VICC and AKI, there was a significant correlation for the proportion of schistocytes by ICSH method for the three ordered groups from AKI KDIGO 1 (n = 1, 1.2% schistocytes), KDIGO 2 (n = 8, median 1.8%, IQR 1.4%‐2.5%) and KDIGO 3 (n = 10, median 4.4%, IQR 2.1%‐8.7%); Kendall's tau *b* correlation coefficient .46, SE .15, *P* = .004.

For the 17 cases with VICC with AKI and > 1.0% schistocytes, 12 (71%) had blood films for examination within 24 hours of snakebite envenoming. Of these, all had ≥1.0% schistocytes on peripheral blood film examination within 24 hours of snakebite envenoming. One case with serial blood films for analysis did not have appropriate time and date labeling to allow timepoints to be determined. The four remaining cases (24%) did not have blood films available for examination within 24 hours of snakebite. Of these, all showed schistocytes greater than 1.0% on the first available blood film examined, ranging between 37 and 72 hours post‐bite.

Of the VICC with no AKI group (n = 104), 30 (30%) had a schistocyte count ≥1.0%. Of these, 27 had complete VICC and three had partial VICC; 12 (40%) had MAHA with thrombocytopenia; 11 (37%) had MAHA without thrombocytopenia; four (13%) had VICC with schistocytes ≥1.0% without anemia or thrombocytopenia, and three (10%) had schistocytes ≥1.0% with thrombocytopenia but no anemia.

## 4 DISCUSSION

We found evidence that a schistocyte count ≥1.0% using the ICSH method is sensitive and specific for differentiating VICC with AKI vs VICC alone. Together with our finding of no AKI in the non‐envenomed and envenomed non‐VICC groups, this supports a hypothesis that TMA is a predominant etiology of AKI in snakebite. This finding is particularly true for Australian elapid envenoming, in which AKI has only rarely been associated with severe myotoxicity and rhabdomyolysis, such as seen with tiger snake (*Notechis scutatus*).^25^

For patients with snakebite VICC we propose schistocytosis ≥1.0% with anemia and thrombocytopenia as diagnostic criteria for TMA in snakebite. This recommendation is consistent with an existing international consensus on schistocyte quantitation threshold for TMA, and with the standardization of terminology for TMA more broadly.^28^, ^29^ If this definition is applied, a small number of outliers will occur, with MAHA and no thrombocytopenia, or thrombocytopenia without anemia, whom are at a much lesser risk of AKI. Similar outliers have been reported in other TMA disorders, such as hemolytic uremic syndrome.^30^, ^31^ We propose that TMA in snakebite is a spectrum disorder. It presents with MAHA and thrombocytopenia, and in a small subset of patients only one of MAHA (with schistocytes >1%) or thrombocytopenia may occur. It would therefore be prudent for patients with snakebite envenoming, VICC and evidence of MAHA with schistocytes ≥1.0%, and/or thrombocytopenia to be carefully observed for AKI with monitoring of urine output and serial creatinine testing.

Our findings regarding the sensitivity and specificity of schistocyte quantitation in snakebite are concordant with existing studies evaluating the diagnostic utility of schistocyte quantitation in other causes of TMA. The existing ICSH consensus guideline recommends a 1% threshold as a robust cut‐off for TMA, concordant with prior evidence regarding schistocyte cut‐offs for the diagnosis of TMA schistocytosis.^13^, ^14^, ^15^, ^28^, ^32^, ^33^, ^34^ Lesesve et al^35^ have reported a 0.5% schistocyte cut‐off when only counting helmet and triangular cells, which gave a 96.2% sensitivity and 93.3% specificity. Abdelkader et al^33^ found a schistocyte cut‐off of ≥0.95% had an 88% sensitivity and 85.3% specificity for prediction of TTP in a population of pregnant women presenting with features of severe preeclampsia and the hemolysis, elevated liver enzymes, low platelet count syndrome, although TTP in this study was determined by clinical criteria not ADAMTS‐13 testing specifically. Our results show nil to minimal schistocytosis in snakebite groups equivalent to normal or negative controls: the non‐envenomed and envenomed cases without VICC. This finding is consistent with other non‐snakebite studies evaluating the degree of schistocytosis in completely normal ambulant populations, which have shown a schistocyte count of less than 0.2%,^13^, ^15^ although their exact method of counting and definition of a schistocyte were not reported. Studies evaluating automated compared to manual methods in schistocyte quantitation have reported similar reference ranges for normal patients, though the automated methods slightly overestimated counts compared to manual examination of the blood film.^36^

Our study has some limitations. The calculation of specificity and sensitivity used the VICC group with AKI as a surrogate for TMA, with the negative control or reference group those with VICC and no AKI. This assumes that AKI is the main end‐organ injury of TMA. We justify this assumption in the context of supportive available literature on snakebite‐associated TMA.^5^, ^6^, ^7^, ^8^, ^9^, ^10^ We had 2/19 cases with VICC and AKI who had a peak schistocyte count of <1.0%. These cases had missing data with no blood films for examination except same day collected specimens. Whether these cases are true TMA with missing data to confirm the schistocytosis, or truly below the 1.0% threshold for significance, or had AKI due to other non‐TMA causes is unknown. Finally, the real‐world application of schistocyte quantitation in global regions where snakebite is most prevalent poses many challenges. These regions may have limited resourcing with respect to laboratory reagents and materials, guidelines, quality control and laboratory scientist training.^37^ Often, only simple bedside testing for the detection of incoagulable blood are available.^3^ Nonetheless, in resource poor settings globally, blood film examination remains central in the diagnosis of malaria and anemia, and provides high yield diagnostic information with relatively simple equipment.^38^ The World Health Organization lists peripheral blood film examination in its model list of essential in vitro diagnostics; and provides written resources with respect to blood film microscopy procedures and morphology bench aids which include schistocyte morphology.^39^, ^40^, ^41^

Our findings underscore the central importance of blood film examination in establishing a diagnosis of TMA. Where available healthcare resources allow, schistocyte quantitation by a trained microscopist will assist in the timely diagnosis of TMA in snakebite, and identification of patients at risk of AKI. In resource limited settings where local resources prohibit blood film examination, we recommend patients with snakebite and features of VICC are observed for a minimum of 24 hours, with serial creatinine testing where possible, and monitoring of urine output. The underlying pathophysiology of snakebite associated TMA remains poorly understood and is a recommended area for future research.

## CONFLICT OF INTEREST

The authors declare no competing interests.

## AUTHOR CONTRIBUTIONS

T Noutsos performed experiments, analyzed results, and made figures and tables. GK Isbister and SG Brown coordinated the recruitment of patients to the study and collected blood films. T Noutsos conceptualized and designed the research under the general direction of GK Isbister. T Noutsos primarily interpreted findings and wrote the paper under the general direction of GK Isbister, BJ Currie, and SG Brown. All authors substantially reviewed, commented on, and approved the final version of the submitted manuscript.

## Supporting information

Figure S1
Table S1

## Data Availability

The data that support the findings of this study are available from the corresponding author upon reasonable request.
