# Supplementary material for: Schistocyte quantitation, thrombotic microangiopathy and acute kidney injury in Australian snakebite coagulopathy [ASP28]
Source: Int J Lab Hematol. 2021 Feb 22;43(5):959–65. doi: 10.1111/ijlh.13497 (PMC8519151; doi:10.1111/ijlh.13497)
Supplement: Supplementary file 1 — Figure S1 Table S1 [file IJLH-43-959-s001.pdf]

Supplementary materials

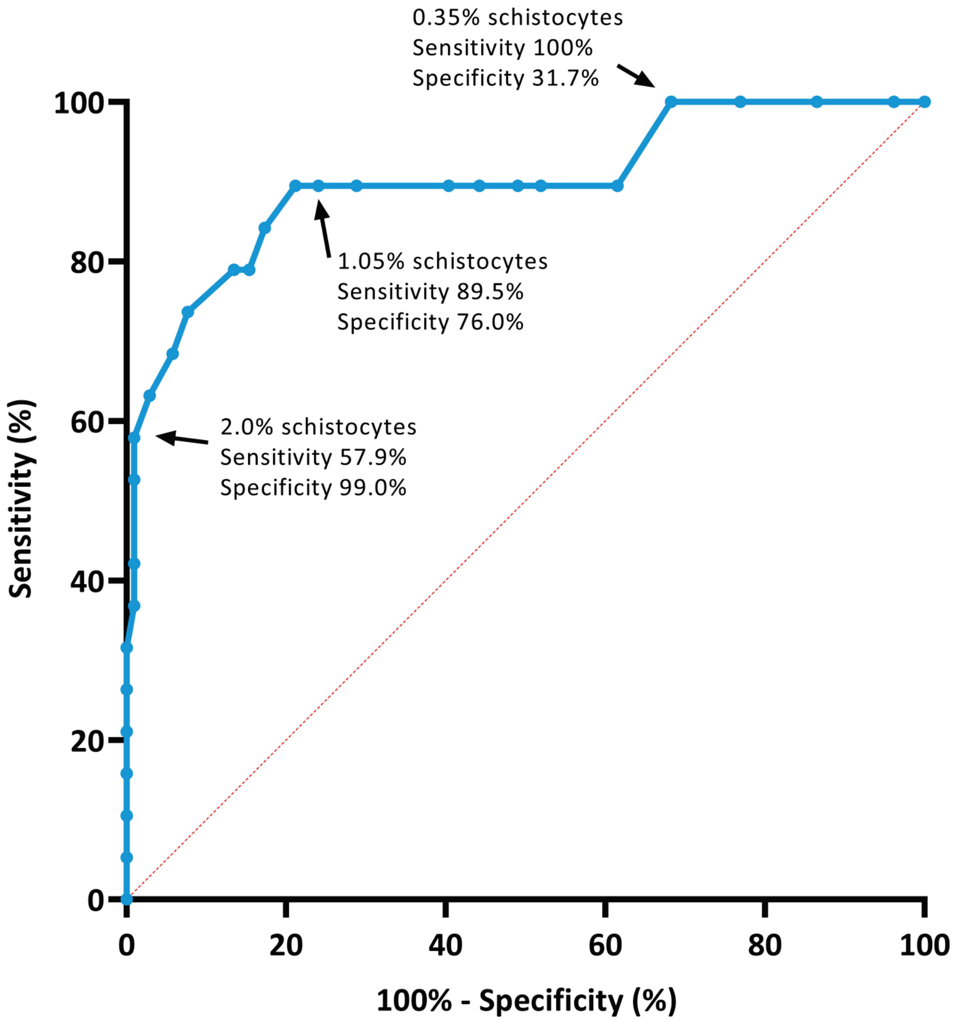

Figure S1. Receiver Operating Characteristic (ROC) curve for International Council for Standardization in Haematology (ICSH) method of schistocyte quantitation for venom-induced consumption coagulopathy (VICC) with acute kidney injury (AKI) versus VICC without AKI

Table S1. Sensitivity and specificity for International Council for Standardization in Haematology method of schistocyte quantitation for venom induced consumption coagulopathy (VICC) with acute kidney injury (AKI) versus VICC without AKI

Schistocytes %	Sensitivity %	95% CI	Specificity %	95% CI	Likelihood ratio
> 0.05	100	83.2% - 100%	3.85	1.51% - 9.47%	1.04
> 0.15	100	83.2% - 100%	13.5	8.19% - 21.3%	1.16
> 0.25	100	83.2% - 100%	23.1	16.0% - 32.0%	1.30
> 0.35	100	83.2% - 100%	31.7	23.6% - 41.2%	1.46
> 0.45	89.5	68.6% - 98.1%	38.5	29.7% - 48.1%	1.45
> 0.55	89.5	68.6% - 98.1%	48.1	38.7% - 57.6%	1.72
> 0.65	89.5	68.6% - 98.1%	51.0	41.5% - 60.4%	1.82
> 0.75	89.5	68.6% - 98.1%	55.8	46.2% - 64.9%	2.02
> 0.85	89.5	68.6% - 98.1%	59.6	50.0% - 68.5%	2.22
> 0.95	89.5	68.6% - 98.1%	71.2	61.8% - 79.0%	3.10
> 1.05	89.5	68.6% - 98.1%	76.0	66.9% - 83.2%	3.72
> 1.15	89.5	68.6% - 98.1%	78.8	70.0% - 85.6%	4.23
> 1.25	84.2	62.4% - 94.5%	82.7	74.3% - 88.8%	4.87
> 1.35	78.9	56.7% - 91.5%	84.6	76.5% - 90.3%	5.13
> 1.45	78.9	56.7% - 91.5%	86.5	78.7% - 91.8%	5.86
> 1.55	73.7	51.2% - 88.2%	92.3	85.6% - 96.1%	9.58
> 1.65	68.4	46.0% - 84.6%	94.2	88.0% - 97.3%	11.9
> 1.75	63.2	41.0% - 80.9%	97.1	91.9% - 99.2%	21.9
> 2.00	57.9	36.3% - 76.9%	99.0	94.8% - 100%	60.2
> 2.35	52.6	31.7% - 72.7%	99.0	94.8% - 100%	54.7
> 2.75	42.1	23.1% - 63.7%	99.0	94.8% - 100%	43.8
> 3.10	36.8	19.1% - 59.0%	99.0	94.8% - 100%	38.3
> 3.65	31.6	15.4% - 54.0%	100	96.4% - 100%	
> 4.80	26.3	11.8% - 48.8%	100	96.4% - 100%	
> 5.55	21.1	8.51% - 43.3%	100	96.4% - 100%	
> 6.75	15.8	5.52% - 37.6%	100	96.4% - 100%	
> 9.50	10.5	1.87% - 31.4%	100	96.4% - 100%	
> 11.3	5.26	0.270% - 24.6%	100	96.4% - 100%	
